# Screening of sleep assisting drug candidates with a *Drosophila* model

**DOI:** 10.1371/journal.pone.0236318

**Published:** 2020-07-29

**Authors:** Yan-Ying Wang, Wei-Wei Ma, I-Feng Peng

**Affiliations:** 1 Research Department, Suzhou Joekai Biotech LLC, Kunshan City, Jiangsu, China; 2 School of Life Science, Tsinghua University, Beijing, China; University of Lübeck, GERMANY

## Abstract

Lately, *Drosophila* has been favored as a model in sleep and circadian rhythm research due to its conserved mechanism and easily manageable operation. These studies have revealed the sophisticated parameters in whole-day sleep profiles of *Drosophila*, drawing connections between *Drosophila* sleep and human sleep. In this study, we tested several sleep deprivation protocols (mechanical shakes and light interruptions) on *Drosophila* and delineated their influences on *Drosophila* sleep. We applied a daytime light-deprivation protocol (DD) mimicking jet-lag to screen drugs that alleviate sleep deprivation. Characteristically, classical sleep-aid compounds exhibited different forms of influence: phenobarbital and pentobarbital modified total sleep time, while melatonin only shortened the latency to sleep. Such results construct the basis for further research on sleep benefits in other treatments in *Drosophila*. We screened seven herb extracts, and found very diverse results regarding their effect on sleep regulation. For instance, *Panax notoginseng* and *Withania somnifera* extracts displayed potent influence on total sleep time, while *Melissa officinalis* increased the number of sleep episodes. By comparing these treatments, we were able to rank drug potency in different aspects of sleep regulation. Notably, we also confirmed the presence of sleep difficulties in a *Drosophila* Alzheimer’s disease (AD) model with an overexpression of human Abeta, and recognized clear differences between the portfolios of drug screening effects in AD flies and in the control group. Overall, potential drug candidates and receipts for sleep problems can be identified separately for normal and AD *Drosophila* populations, outlining *Drosophila’s* potential in drug screening tests in other populations if combined with the use of other genetic disease tools.

## Introduction

Health has long been associated with adequate sleep quality and sleep quantity: studies have shown that an average sleep duration of 7 hours per night is strongly correlated to an improvement in human health [1]. However, sleep problems affect nearly one in seven adults, especially individuals who regularly travel across time zones or have diseases including but not limited to Alzheimer’s disease and schizophrenia [2,3]. Melatonin is commonly used as a food supplement to treat jet lag related sleeping problems, but a number of studies have reported that it merely decreases sleep latency and does not increase total sleep episodes or sleep quantity [4–6]. Moreover, although sedative-hypnotic drugs such as pentobarbital and phenobarbital have also been used as sleep enhancers for treating insomnia, adverse effects such as potential drug dependence and drowsiness have limited their usage [7–9]. As a result, there is an unmet need for sleep enhancers without strong adverse effects in both scientific research and commercial applications.

*Drosophila* is an excellent model for human behavior, because it exhibits many different types of humanlike conduct, including fighting, learning and memory, and drug addiction [10–12]. Flies are also extensively used in circadian research: the first circadian gene, *Per*, was first discovered in *Drosophila* [13]. In recent years, *Drosophila* has been favored as a model in the discovery of treatments regulating sleep and circadian rhythm, used parallel to conventional mouse and large animal models; key sleep parameters have been defined, and multiple different types of sleep deprivation (SD) approaches have been applied [14–16]. These approaches can be categorized into the following: sensory stimulation, circadian clock alteration, and genetic manipulation. Each SD stimulation approach has its own advantages and limitations. For example, physical vibrations can inflict significant levels of change in sleep, but can also cause non-specific side effects [16]. While genetic activation of circadian neurons has minimal effects on sensory stimulation, it may also reduce the life span of flies [17]. Therefore, different SD protocols must be adopted and modified to fulfill specific needs in different settings. In addition, herbs extracts have been used extensively in managing jet lag and disease related insomnia in Eastern Asia [18,19]. However, only a small percentage of these extracts have been tested in animal models, and an even lesser percentage have been tested in official clinical trials [20,21]. Therefore, developing a fly-based assay can provide a platform for evaluating distinct sleep parameters.

In this study, we used the *Drosophila* Activity Monitoring System (DAMS) to take time-lapse recordings of daily fly activity. Multiple SD protocols mimicking different biological scenarios were examined and compared. We selected a daytime light deprivation protocol (Dark-dark daily cycle, DD) mimicking jet lag to conduct drug screening and create profiles for sleep regulation. Additionally, we utilized a *Drosophila* Alzheimer’s disease (AD) model overexpressing human Abeta peptide in the central nervous system [22–24] in the screening process, and found that the screening portfolios of the herbal extracts were quite different from control files. Viewed in junction, potential drug candidates and receipts for sleep problems can be identified separately for control or AD *Drosophila* populations, and also in other populations if combined with the use of other genetic disease tools.

## Materials and methods

### Flies

The isogenic line of w1118 (isoCJ1) was used as a control in all experiments and is referred to as control flies. The expression of human Ab42 peptide in *Drosophila* was performed by a genetic cross of the UAS-hAbeta42 line and the elav-GAL4c155 line (an ubiquitous neuronal expressing Gal4 line), and the offspring strain will be referred to as AD flies [22–24]. All flies were reared at 25 ºC and 50% relative humidity in a 12-hour light and 12-hour dark cycle (LD) condition.

### Sleep assays

On the first day after eclosion (DAE), virgin male or female flies were identified and sorted separately by their gender in vials (3 cm in inner diameter) with a standard cornmeal medium for recovery. From DAE 2 to 4, flies were raised in a sucrose-agar (SA) medium (4% and 1%, respectively) containing either the vehicle or drugs. At the end of DAE 4, each fly was transferred into individual recording tubes (0.3 cm in inner diameter) with the same type of SA medium, and was then acclimated in an incubator for at least 24 hours in the LD condition (adaptation stage). The settings for lights on and off were at Zeitgeber (ZT) 12 (local time 20:00) and ZT 24(0) (local time 08:00), respectively. After turning off the light on DAE 5, data collection was performed using the DAMS (Trikinetics Waltham, MA) for one to three days with or without SD protocols (see next section). Events are recorded when a fly crosses the detection point and the intervals between consecutive events is 6 seconds. Based on earlier publications, 5 minutes of consolidated inactivity is defined as sleep in *Drosophila* [14–15]. We used Matlab (MathWorks, Natick, MA) to process raw data from the DAMS to obtain individual sleep parameters for each 12 hour period. Sleep parameters were adopted from previous studies [25]: total sleep time, the total sleep duration in given period; sleep latency, the time period to first sleep after lights off (ZT 12); activity index, the frequency of activity during waking time; mean sleep, the mean duration for sleep episodes; maximum sleep, the duration for the longest sleep episode; sleep frequency, the number of sleep episodes in a given period.

### Protocols for sleep deprivation

SD protocols used in this study include the discontinuous mechanical stimulation (DMS), the discontinuous light stimulation (DLS), and the constant darkness (DD) condition. For DMS, flies were subjected to mechanical disturbance with a shaker (a 5-sec shake, about 2-cm shift at 240 round/min, with a cycle of every 30 min) for 12 hours during the night (ZT 12–24). DLS is a method adapted from a previous publication [26]. Briefly, flies were exposed to 10-min light (490~590 lux) every 60 min for 12 hours during the night. For DD, flies were subjected to constant darkness during the day (ZT 0–12) [16]. For all SD experiments, a group of flies in LD without sleep interference was observed at the same time as a control group.

### Drug treatment and preparation

Drugs, extracts or vehicles were added to fly food since 2 DAE. Sources and final concentrations in SA medium for three compounds are as following: pentobarbital was obtained from Sigma-Aldrich Co. (St. Louis, MO, USA) at 1 mg/ml; phenobarbital was obtained from New Asiatic Pharmaceutical (Shanghai, China) at 720 ug/ml; melatonin was obtained from Meilun Biotech. Co. (Dalian, Shenyang, China) at 1 mg/ml. 100X stock solutions were prepared in distilled water (pentobarbital and phenobarbital) or DMSO (melatonin). Seven extracts with known effects on brain function were also selected in the study, as summarized in Table 1. Herb extracts were dissolved at a final concentration of 1 mg/ml in the SA medium.

**Table 1:** Information for herb extracts.

Herbs	Parts Used	Extract Method	Extract Ratio	Vendors	Lot No.	Concentration	Published Function
*F*. *multiflora*	NS^a^	water	10: 1 (w/w ^b^)	Ruikang Bio. Eng.	20171115	1 mg/ml	Sedation and hypnosis [43]
*G*. *uralensis*	NS ^a^	water	10: 1 (w/w ^b^)	Ruikang Bio. Eng.	20171220	1 mg/ml	Cognition related [44]
*M*. *officinalis*	Whole	EtOH & water	10: 1 (w/w ^b^)	Jiahe Phytochem	CXFC-A-801022	1 mg/ml	Emotion & sleep related [21, 41, 42]
*P*. *ginseng*	Root	EtOH	ginsenosides = 10%	Hongjiu BioTech	160920	1 mg/ml	Cognition & anti-inflammatory [45]
*P*. *notoginseng*	Root	EtOH & water	saponin ≥ 30%	Jiahe Phytochem	Csq20151019	1 mg/ml	Emotional related [19, 36–39]
*P*. *vulgaris*	NS ^a^	water	10: 1 (w/w ^b^)	Ruikang Bio. Eng.	20171011	1 mg/ml	Sleep related [46]
*W*. *somnifera*	Root	EtOH & water	withanolides ≥ 2.5%	Jiahe Phytochem	CZQ-A-701094	1 mg/ml	Sleep related [47]

^a^NS: not specified.

^b^w/w: weight/weight.

### Analysis and statistics

In most figures, data is presented as mean ± standard error of the mean (SEM). “N” represents the number of flies tested. Unpaired two-tailed Student’s *t*-test was used for statistical analysis (GraphPad 7, GraphPad Software, San Diego, CA). p < 0.05 was considered to have a significant difference. *, p < 0.05; **, p < 0.01; ***, p < 0.001; ****, p < 0.0001. In Figs 4, 5 and 7, and S2 Fig, data is presented as the differences of individual groups from the control group. This normalization was performed so that the potency of specific parameters among groups could be ranked. One-way ANOVA followed by a Fisher’s LSD test as the post-hoc test was used for statistical analysis, conducted through GraphPad 7. Similarly, *, p < 0.05; **, p < 0.01; ***, p < 0.001; ****, p < 0.0001.

## Results

### Different SD protocols show a distinct effect on *Drosophila* sleep parameters

**Fig 1:**
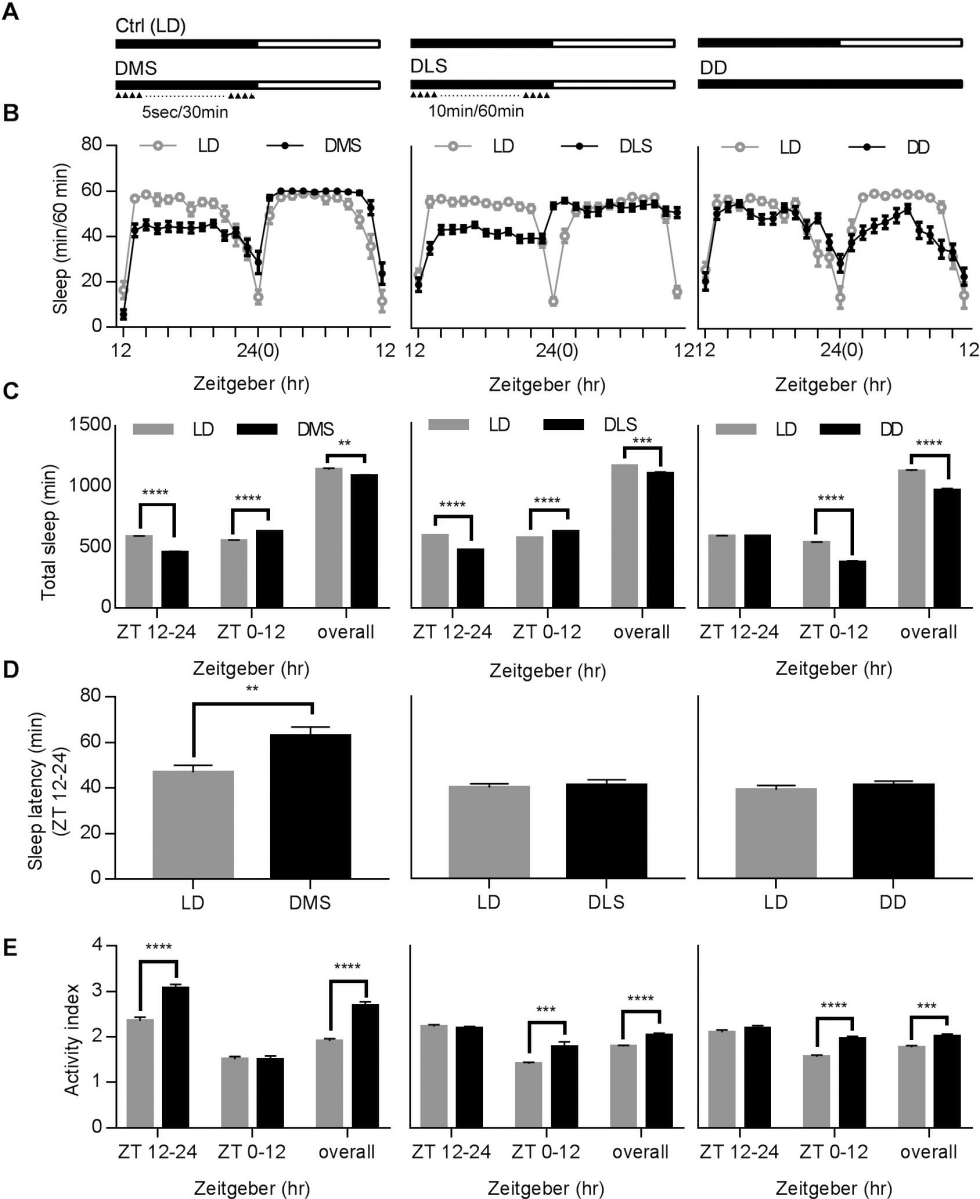
Distinct effects of three different sleep deprivation (SD) protocols in *Drosophila*. (A) Schematics for three SD protocols. Black and white bars indicate the lights-off and lights-on duration respectively. Triangle symbols indicate the delivery of mechanical or light stimuli, and dot lines represent the omitting stimuli. Ctrl (LD): Control Light-Dark Condition; DMS: Discontinuous Mechanical Stimulation; DLS: Discontinuous Light Stimulation; DD: Dark-Dark Stimulation. (B) Typical sleep profiles of manipulated (DMS, DLS, DD) and control (LD) flies. Male flies were used in this test. (C) Comparison of total sleep time between the control group and three different SD protocols in different time periods. (D) Comparison of sleep latency (latency to the first recording of sleep after lights off) between the control group and three different SD protocols. (E) Activity index affected by different SD protocols. Data are presented as mean ± SEM. Tested fly N numbers: Left column, 48 for LD and 48 for DMS; middle column, 172 for LD and 156 for DLS; right column, 102 for LD and 105 for DD. Two-tailed, unpaired Student’s t-test was used. **, p < 0.01; ***, p < 0.001; ****, p < 0.0001.

Sleep in *Drosophila* can be disrupted by many environmental factors, including but not limited to vibration, sound, and light. Several sleep-deprivation protocols were designed to mimic different scenarios in daily activity [14,15,26]. We used a DAMS designed for time-lapse recording of daily fly activity to study the effects of different factors on the sleep profiles of flies. Three sensory stimulation-based approaches were tested in this study, including vibration-based DMS and light interference-based DLS and DD protocols (Fig 1A, also see Materials and Methods). DMS and DLS were designed to model the effects of disturbance at night time, and DD models changing work shifts between day and night or shifting between time zones, mimicking jet lag in humans. Fig 1B depicts the separate sleep profiles of male flies, corresponding to their individual stimulations. Compared to the sleep profiles of the control group, flies under DMS and DLS experienced a reduction in sleep at night (ZT12-24), while the ones in the DD condition exhibited a strong reduction mainly at ZT 0–12 and not at ZT 12–24. Statistical analysis shows that flies after DMS have 12% more total sleep at ZT 0–12 and an increase in sleep latency (Fig 1C and 1D, left panel). On the other hand, flies after DLS exhibit no significant changes in total sleep time during ZT 0–12 and sleep latency (Fig 1C and 1D, middle panel). It is worth noting that the activity index (activity count/wake time) can also be obtained through our methods, illustrating basic fly activity behaviors during waking period. In many other animal models like rodents, additional experiments must be carried out for such information. According to our results, activity level was affected in an opposite way by mechanical and light stimuli during period ZT 12–24 (Fig 1E, DMS up and DLS down, respectively), and might affect the following period ZT 0–12 only in DLS. Contrastingly, DD protocol increased the activity level at ZT 0–12 (Fig 1E).

**Fig 2:**
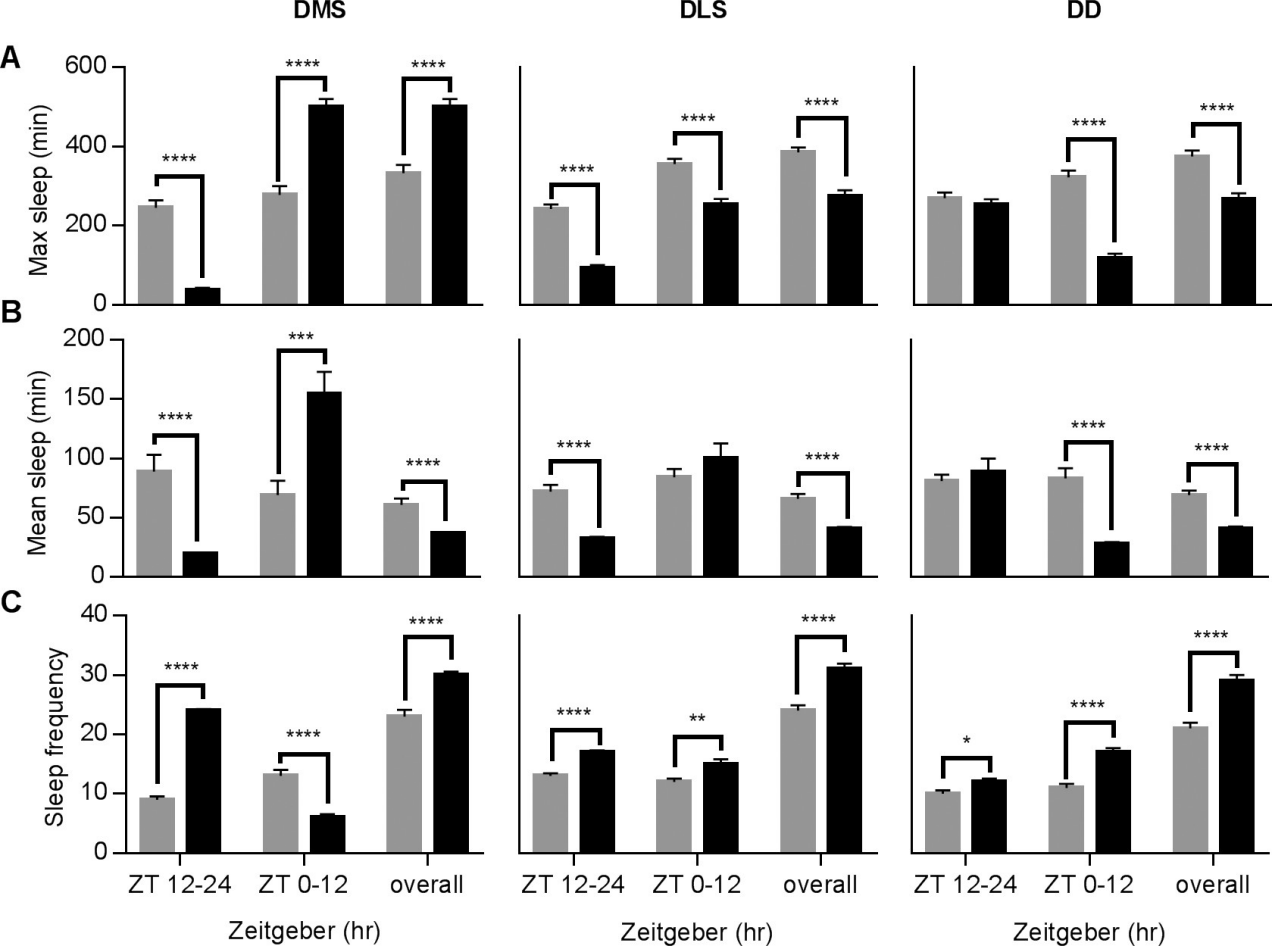
Additional sleep parameters changed in three different SD protocols. Additional sleep parameters from the control LD and corresponding experiment groups were illustrated with gray and black bars, respectively. (A) The effect of three SD protocols on maximum sleep episode duration during SD. (B) Mean sleep episode duration during SD. (C) The effect of DLS, DMS and DD on sleep frequency (number of sleep episodes in fix time). N numbers and statistical methods are the same as in Fig 1.

This time-lapse recording system also helps reveal several complicated parameters in the sleep profile rarely obtained in other animal models. For instance, we were able to record maximum and mean sleep length and sleep frequency, three other indicators of sleep quality. These parameters experienced significant changes (Fig 2): DMS reduced maximum and mean sleep lengths and increased sleep frequency (ZT12-24), and exhibited significant rebound in maximum and mean sleep after SD (ZT 0–12). Contrastingly, the DLS group displayed phenomena opposite to the DMS group after SD (ZT 12–24), indicating that the rebound period of sleep profiles after light and mechanical stimuli are quite different.

### Different genders showed difference sleep behavior in DD

**Fig 3:**
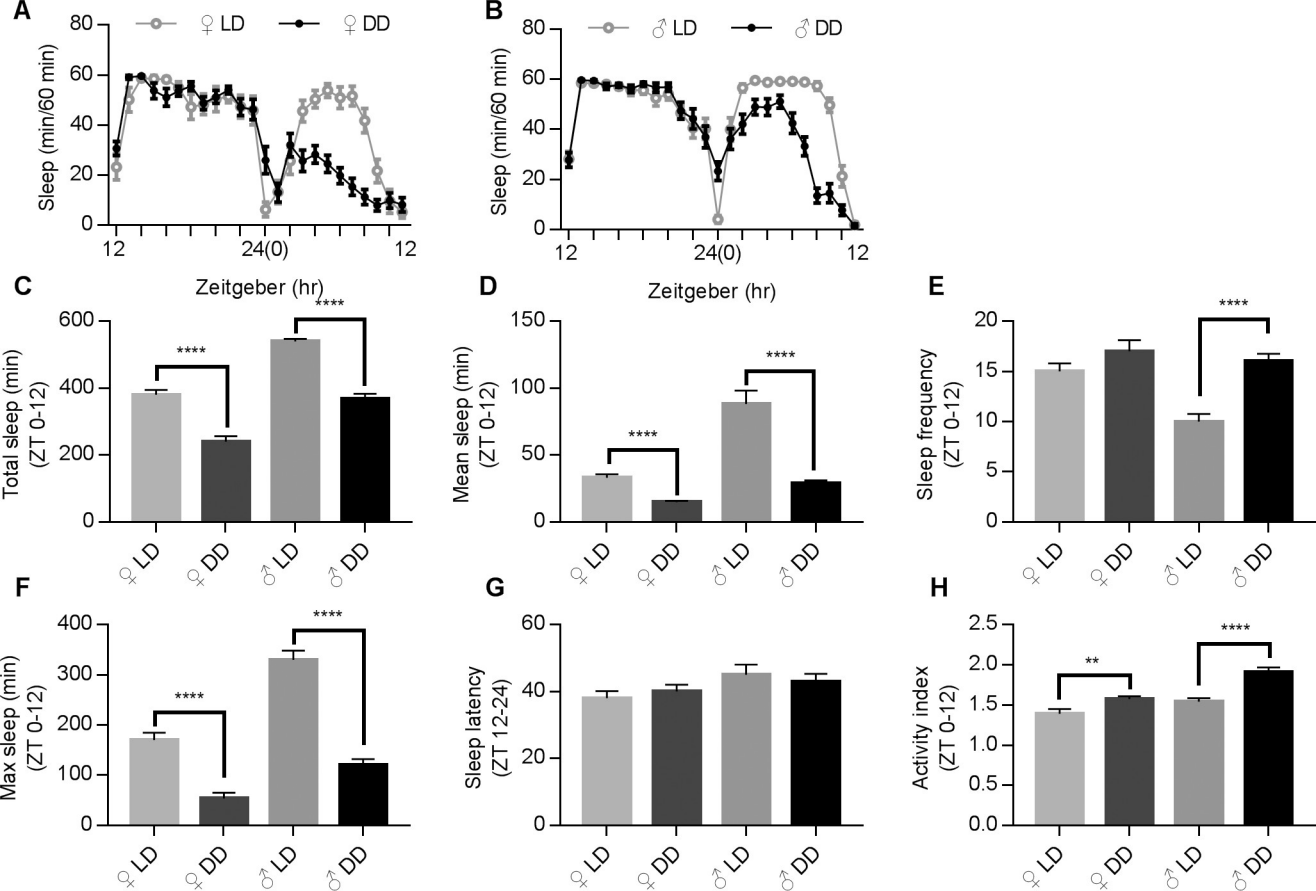
Female and male flies exhibiting distinct sleep profiles in LD and DD conditions. (A-B) Typical sleep profiles for female (A) and male (B) in LD and DD conditions. (C-H) Comparison of sleep parameters under DD and LD conditions between different genders. Tested fly N numbers: female, 78 for LD and 83 for DD; male, 86 for LD and 89 for DD. Statistical methods are the same as in Fig 1.

In order to address sleep-deprivation issues related to changing work shifts and jet lag, we applied the DD protocol for the rest of this study. We first obtained the sleep profiles for both male and female flies to address potential gender differences in circadian rhythm, a phenomenon that had been previously reported in numerous species and also humans. Virgin flies were chosen so that the egg laying would not cause significant difference to the sleep profiles. Our results indicated that female and male flies exhibited discrete sleeping profiles (as shown in Fig 3A and 3B). Compared to male flies, females tend to have less sleep time at ZT 12–24 in regular LD condition. After the DD protocol was applied, female files experienced more reduction in total sleep than male flies (female vs. male, 37.4 + 3.9% vs. 31.5 + 2.5%, respectively, Fig 3C), and this tendency of reduction continued in the following days (S1 Fig). Both female and male flies experienced a reduction in mean and maximum sleep length (Fig 3D & 3F). Sleep frequency was increased about 70% in males during DD, much larger in comparison to the change in females (Fig 3E). We concluded that changes induced by DD in sleep latency were not significant (Fig 3G). However, male flies did have a significantly higher activity index (Fig 3H). Overall, female flies were more vulnerable to DD stimulation considering total sleep time, but not in the sleep frequency. Therefore, female flies were chosen for the following drug screening study.

### Drug screening using DD paradigm in *Drosophila*

**Fig 4:**
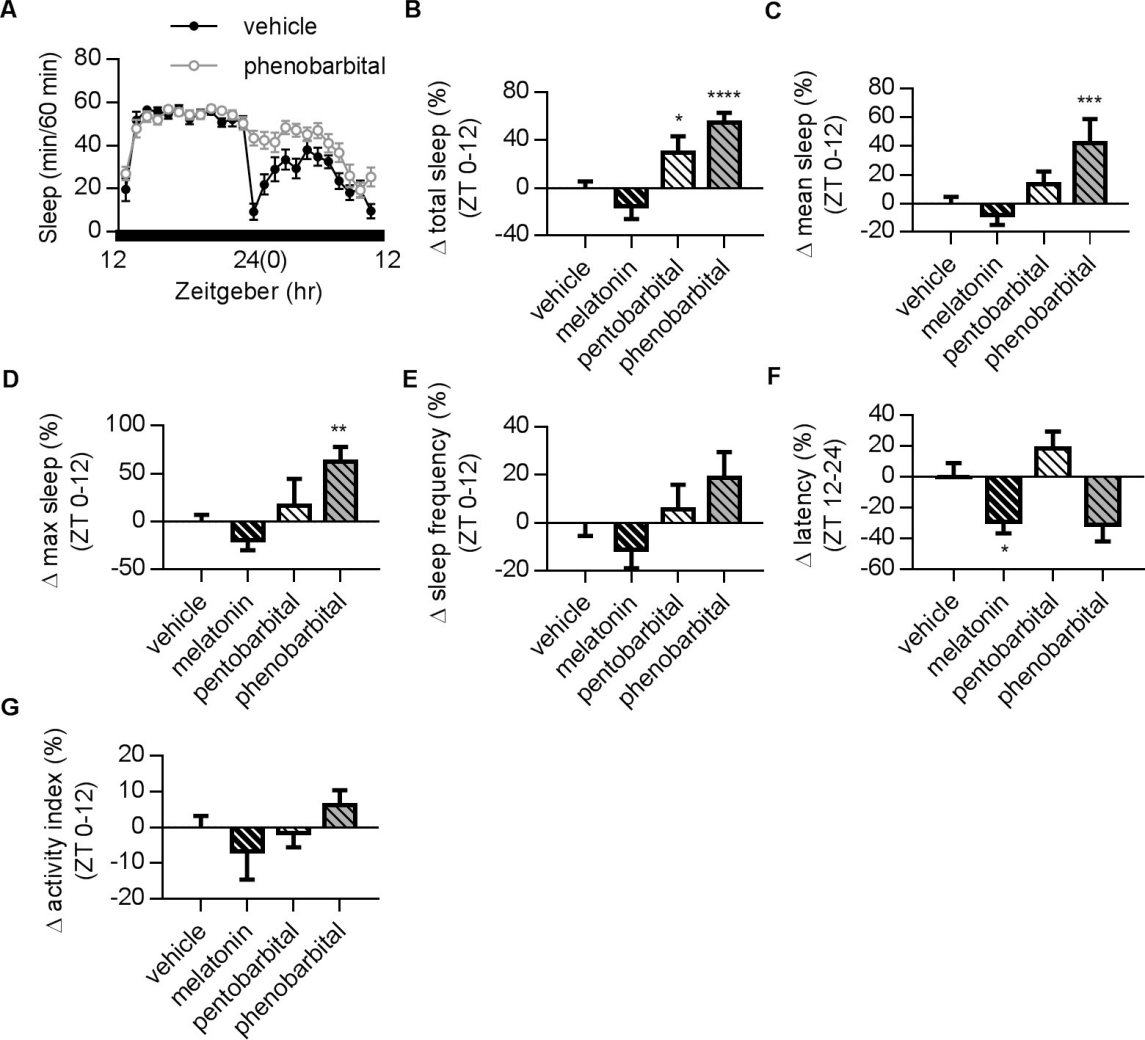
Effects of different sleep-aiding compounds in DD condition. (A) Representative plots showing the sleep profiles of vehicle and phenobarbital in DD conditions. (B) Effects of melatonin, pentobarbital and phenobarbital on total sleep time at ZT 0–12. Drugs were presented in food three days before and during the recordings. The results for ZT 12–24 are shown in S2 Fig. Phenobarbital and pentobarbital significantly increased total sleep. (C) Summary of changes on mean sleep time. Phenobarbital significantly increased mean sleep. (D) Summary of changes on maximum sleep duration. Phenobarbital significantly prolonged maximum sleep. (E) Three drugs had no significant effect on sleep frequency. (F) Melatonin significantly reduced latency. (G) Activity index remained unchanged in all drug-treatment groups. Values for individual groups illustrate the changes in percentage from vehicle group after normalization for comparisons in (B-G). Error bars indicate SEM. N numbers for vehicle, melatonin, pentobarbital, and phenobarbital: 40, 15, 16, and 12. One-way ANOVA followed by a Fisher’s LSD test (compared with the vehicle feeding group) was used for statistical analysis. *, p < 0.05; **, p < 0.01; ***, p < 0.001; ****, p < 0.0001.

Due to its easily manipulated operation, it is possible to perform phenotypic screening for drugs in *Drosophila* [24,27,28]. In order to determine if the DD protocol is suitable for drug screening, we tested several common medications prescribed for sleep therapy. In Fig 4, the differences of drug-given groups from the control group were presented so that the potency at specific parameters among groups could be ranked. We found that phenobarbital, a widely used sleep enhancer, significantly increased total sleep, mean sleep, and maximum sleep at ZT 0–12 (Fig 4A–4E). On the other hand, pentobarbital only resulted in a slight increase on total sleep time (Fig 4B–4G). It is worth noting that the complicated parameters retrieved by time-lapse recordings from large samples enable more sophisticated analysis of drug effects on sleep profiles. It is possible to delineate the therapeutic effects of drugs with similar structures with the *Drosophila* system. The exogenous melatonin administration has been previously documented to improve human circadian rhythms and sleep [5,6]. Our results reveal that melatonin treatment only reduces latency in flies (Fig 4F) and has limited effects on other sleep parameters (Fig 4B–4G).

**Fig 5:**
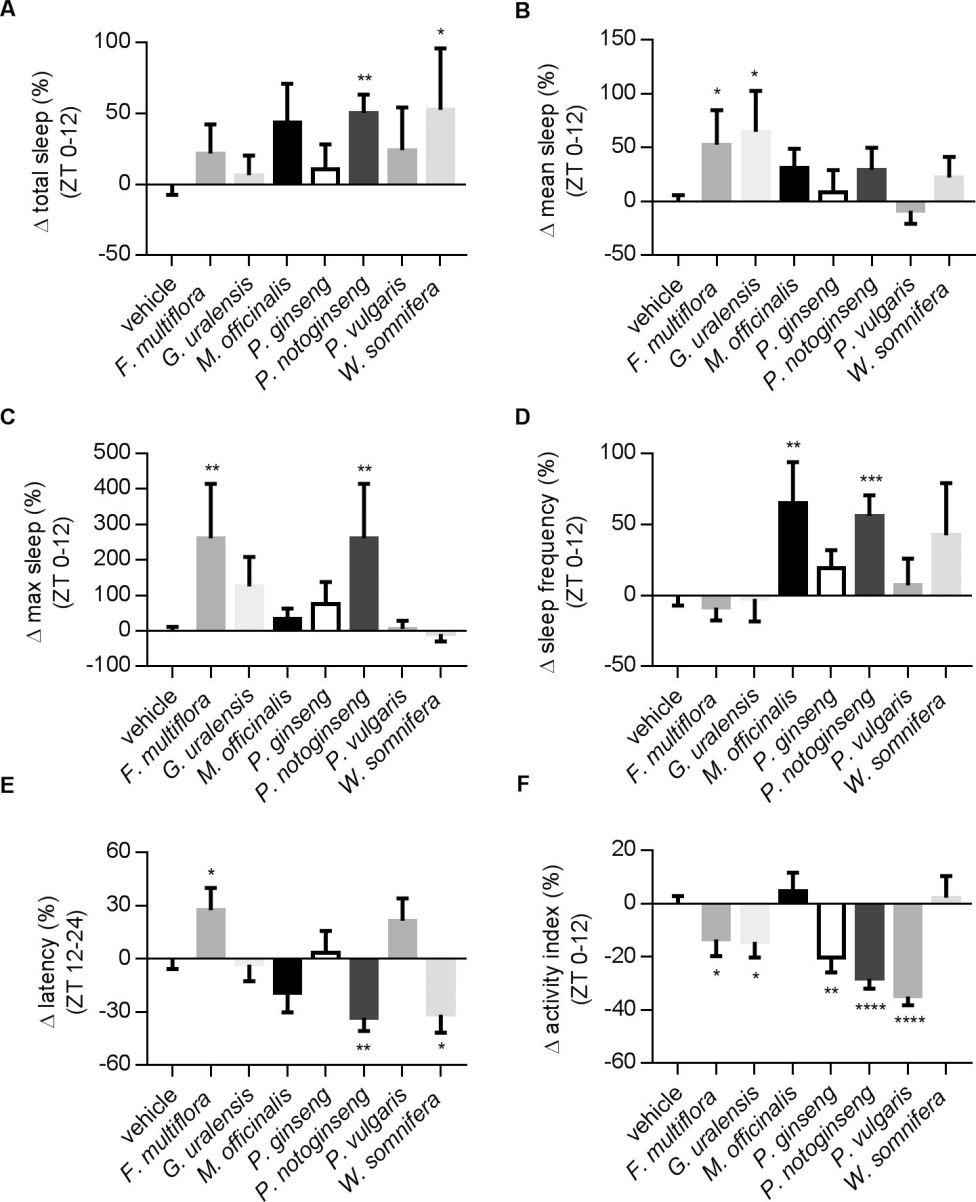
Effects of herb extracts in DD. A summary of seven herb extracts for their effects on sleep parameters. Drugs were presented in food three days before and during the recordings. (A) Changes in total sleep time. *P*. *notoginseng* and *W*. *somnifera* significantly increased total sleep. (B) Changes in mean sleep duration. *F*. *multiflora* and *G*. *uralensis* significantly increased mean sleep. (C) Changes in maximum sleep. *F*. *multiflora* and *P*. *notoginseng* prolonged the maximum sleep. (D) Changes in sleep frequency. Flies fed with *M*. *officinalis* or *P*. *notoginseng* had more sleep episodes. (E) Sleep latency. *P*. *notoginseng* and *W*. *somnifera* decreased sleep latency. (F) Changes in activity index. Except for *M*. *officinalis* and *W*. *somnifera*, all other drugs reduced activity index. The concentrations of all herb extracts were 1 mg/ml in food. Values for individual groups illustrate the changes in percentage from vehicle group after normalization. Error bars indicate SEM. N numbers: vehicle, N = 77; *F*. *multiflora*, N = 26; *G*. *uralensis*, N = 14; *M*. *officinalis*, N = 13; *P*. *ginseng*, N = 14; *P*. *notoginseng*, N = 28; *P*. *vulgaris*, N = 15; *W*. *somnifera*, N = 13. Statistical methods are the same as in Fig 4.

Many herbs like *F*. *multiflora*, *G*. *uralensis*, *P*. *notoginseng*, and *M*. *officinalis* have been documented to modulate the function of central nervous system or to relieve sleep disturbances in rodents and other species [8,19,21,29, 43–47]. We launched a screening of these herbs (see Table 1) to examine their therapeutic effect on *Drosophila* sleep profiles. The capacity of DAMS can be expanded by connecting multiple devices in parallel, thus allowing us to test many drug samples simultaneously. An experiment testing seven drugs and one control group (Fig 5) can be finished within days, demonstrating the efficiency of the *Drosophila* system in pharmacological screening. Fig 5 showed that at a 1 mg/ml concentration in daily food, only a few herbal extracts could significantly affect the sleep profile. For example, treatments of *P*. *notoginseng* make flies increase total sleep and decrease latency, but they also reduce fly activity during wake time (Fig 5A, 5E and 5F). The effects of *W*. *somnifera*, also known as Ashwagandha, are very similar to *P*. *notoginseng*, with the exception of its activity index, which remains consistent (Fig 5A, 5E and 5F). On the other hand, *M*. *officinalis*, *P*. *ginseng* and *P*. *vulgaris* exhibit no significant changes on total sleep time, mean sleep, maximum sleep, and latency (Fig 5). Interestingly, *P*. *ginseng* and *P*. *vulgaris* both significantly reduce the activity index before and during SD (Fig 5F and S2 Fig), implying their potential as sedative compounds in our system.

### Sleep profiles in *Drosophila* Alzheimer’s disease models and potential drug screening

**Fig 6:**
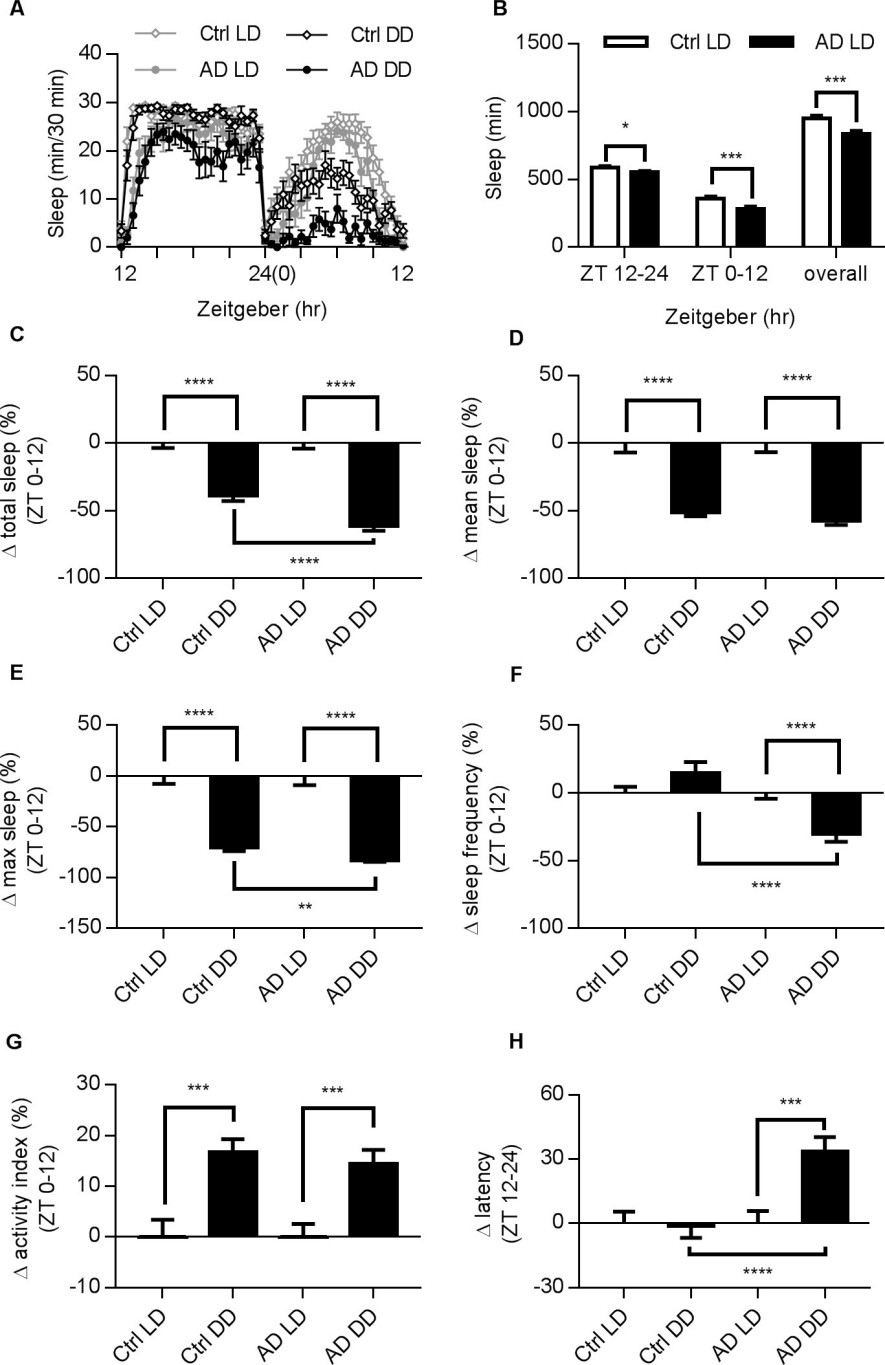
Altered sleep profiles in AD female flies. (A) Typical sleep profiles of female AD and control files. AD flies tended to have shorter sleep in both LD and DD conditions. Male AD flies also have altered patterns (see S3 Fig). (B) Summary of total sleep time for both groups in specific time periods. (C-H) Comparison of LD and DD conditions in both strains and changes between strains under DD. DD data is presented against corresponding LD groups after normalization. Error bars indicate SEM. (C) Changes in total sleep: both groups of flies exhibited reduction in total sleep time in DD, but such change was greater in the AD group. (D) AD and control flies exhibit similar level of reduction in mean sleep. (E) AD flies had more reduction in maximum sleep. (F) Significant differences in the sleep frequency could be detected only in AD flies. (G) Both strains were more active under DD. (H) AD flies showed longer sleep latency. N numbers for control flies in LD, control flies in DD, AD flies in LD, and AD flies in DD: 90, 95, 87, and 87. Statistical methods are the same as in Fig 1.

AD patients exhibit not only cognitive deficits but also dysfunction of the circadian rhythm and other sleep disturbances [30,31]. To investigate the phenotypes on sleep caused by AD in *Drosophila*, we used an AD model, elav-Gal4 x UAS-hAbeta-42, which has an overexpression of the human-A beta 42 peptide in the central nervous system (see Materials and Methods) and has been shown to exhibit progressive cognition deficits and neurodegeneration [22–24]. In this study, we first reported altered sleep profiles in AD flies in both female (see Fig 6A) and male (S3 Fig) flies in a regular LD cycle. The AD flies also exhibited a decrease in total sleep time in female (Fig 6B) and male (S3 Fig), similar to the trends found in earlier clinical studies. Under the DD condition, AD flies exhibited reduced change rate in total sleep time, maximum sleep, sleep frequency, and increased latency as compared to control flies (Fig 6C–6H). Notably, control and AD flies showed similar changes in activity index (Fig 6G). Overall, AD flies have sleep issues and are more vulnerable to DD condition (Fig 6 and S4 Fig).

**Fig 7:**
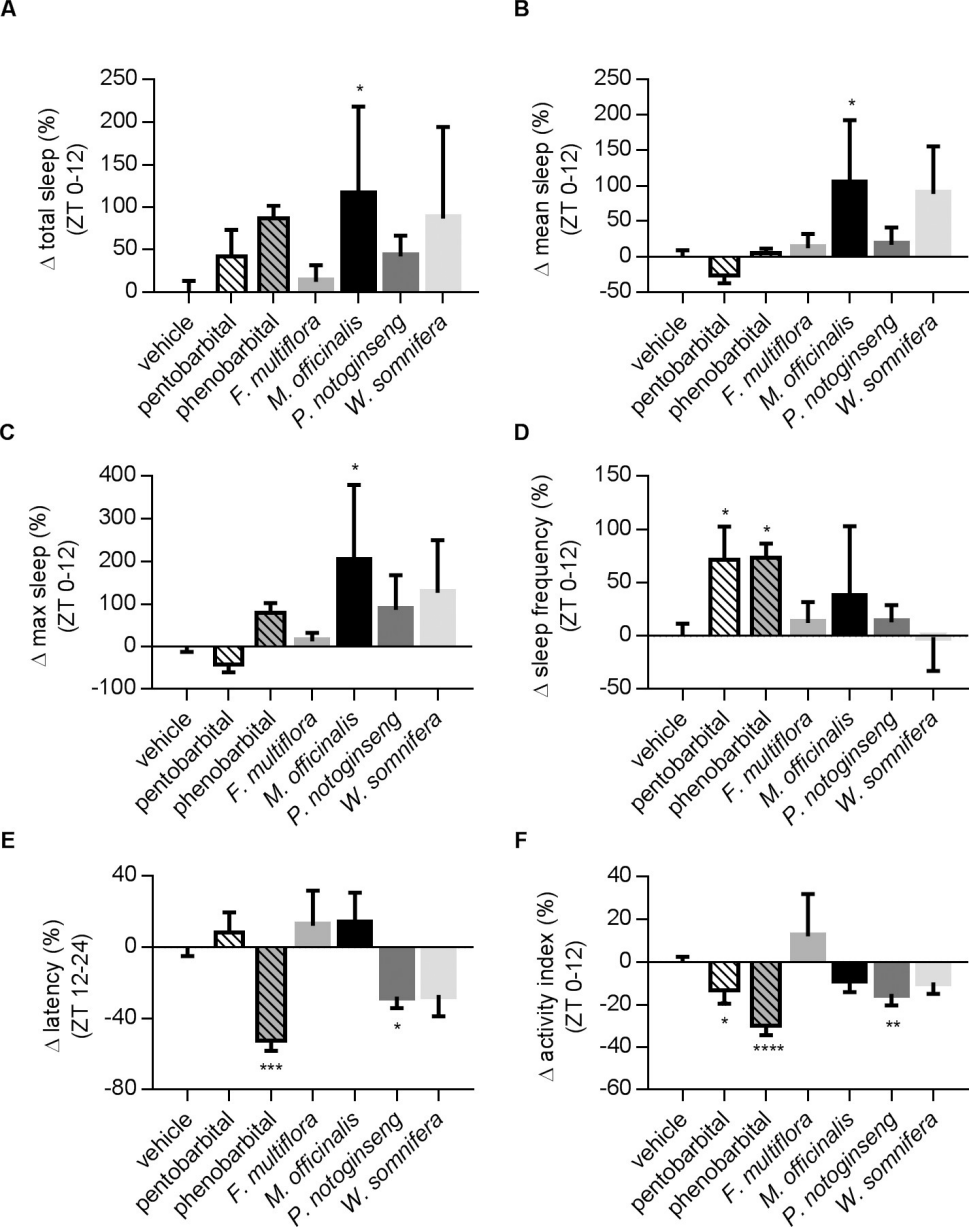
Portfolios of drug effects on AD flies in DD. The effects of two compounds and four herbal extracts were tested in female AD flies. The changes of individual parameters were normalized against the vehicle group. (A) Total sleep: *M*. *officinalis* significantly increased total sleep. (B) Mean sleep: *M*. *officinalis* significantly increased mean sleep. (C) *M*. *officinalis* significantly prolonged maximum sleep. (D) AD fed with pentobarbital or phenobarbital had more sleep episodes. (E) Only phenobarbital and *P*. *notoginseng* could reduce sleep latency in AD flies. (F) *F*. *multiflora*, *M*. *officinalis* and *W*. *somnifera* had no effects on activity in AD flies. N numbers: vehicle, N = 86; pentobarbital, N = 16; phenobarbital, N = 14; *F*. *multiflora*, N = 31; *M*. *officinalis*, N = 15; *P*. *notoginseng*, N = 27; *W*. *somnifera*, N = 15. Statistical methods are the same as in Fig 4.

It is interesting that the drug screening results in control and AD flies are quite different (Figs 4, 5 and 7). Pentobarbital and phenobarbital have no significant effects on many of the sleep parameters in AD flies, but they both significantly suppress the activity index and lead to a strong reduction in activity during wake time (Fig 7A–7F). Among the herbal extracts, we found that extracts from *M*. *officinalis* enhanced the total sleep, mean sleep and maximum sleep time of flies (Fig 7A–7C).

## Discussion

Sleep regulation involves complex mechanisms in all animal species. Environmental factors such as a change in light and chemical stimulants like caffeine can modulate the sleep patterns in *Drosophila* [14,15,32]. Therefore, *Drosophila* has a high potential in the screening of drug treatments for human use. For many years the sleep phenotypes of *Drosophila* have been used in genetic screens to isolate dozens of mutants, providing valuable insight into the molecular mechanisms of sleep behavior for both invertebrates and vertebrates. Drug screens in *in vivo* systems are rare, especially when behavioral outputs such as sleep are involved. There are only a few studies reporting *in vivo* screening for sleep phenotypes in small animals like *Zebrafish* and *Drosophila* [27,33]. Several automated, sophisticated setups have been developed recently not only to reduce labor-intensive experiments but also to gain detailed information about sleep architecture. In this study, we chose the DAM system (see Materials and Methods), which allows multiple drug screening tests simultaneously. Since these drugs are tested within a short period of time, their pharmacological results can be directly compared to all others (Figs 4, 5 and 7, and S2 Fig), producing results of high efficacy.

*Drosophila* sleep shares many key characteristics with mammalian sleep. In humans, sleep is a dynamic physiological process involving multiple transitions between the rapid eye movement (REM) stage and three other non-REM stages, each associated with different arousal thresholds [34]. In *Drosophila*, total sleep duration is often equated with sleep intensity [14–15]. Interestingly, a recent publication used electrophysiology and arousal-testing methods to identify dynamic deep sleep stages in *Drosophila* [35]. By considering how sleep profiles in dynamic stages may change after behavioral and pharmacological manipulation or in mutant strains, we can uncover the functional roles of specific parameters in sleep processes. In this study, we monitored changes on several important parameters, including total sleep time, sleep latency (time to sleep) and activity index (locomotion counts/wake time), revealing their different influences on the *Drosophila* sleep profile. For instance, the administration of melatonin shortened sleep latency but did not increase the total sleep time during sleep deprivation (Fig 4A, 4B and 4F), indicating that melatonin may only induce flies to sleep faster but not longer. Such results are consistent with human clinical trials that have reported that the prescription of melatonin helps induce sleep but does not enhance total sleep time [4]. Moreover, the activity index shows no change after melatonin treatment (Fig 4G), suggesting that melatonin has a limited effect on activity during wake time.

In AD, 44% of patients are affected by sleep disorders, and the prevalence and severity of sleep disturbances increases with AD severity [40,48,49]. With the progression of dementia, the neurons in the suprachiasmatic nucleus (SCN) responsible for the circadian cycle becomes pathologically degenerated [50–52]. Some mouse models with triple-transgenic manipulations can recapitulate many features of AD patients, including reduced activity and similar neuropeptidergic change in the SCN after the onset of Abeta pathology [53]. In AD flies, the expression of Abeta in the brain results in neuronal loss in neuropil regions and the Kenyon cell layer [22] and affects sleep profiles (Fig 6, S3 Fig and S4 Fig). Independently, increasing the sequential cleavage of amyloid precursor protein by expressingβ-site secretase enzyme (dBACE) in fly brains caused the disruption of the circadian rhythm, accompanied by defected oscillation of the PER protein in master regulator neurons in *Drosophila* [54]. These observations suggest a conserved modulation of sleep levels by AD-related proteins across different species, presumably allowing us to use these models for drug discovery. As illustrated in Figs 5 and 7, the drug pharmacology tests in control and AD flies are slightly different. For instance, the treatment of *P*. *notoginseng* can increase total sleep in control flies but not in AD; while the extract from *M*. *officinalis* has limited effects on control flies but greatly enhances total sleep time in AD. It is reasonable to speculate that sleep regulation in control and AD populations may involve different factors and targets in different pathways, although this hypothesis requires further support from studies involving in-depth analysis of molecular mechanisms.

## Supporting information

S1 Fig Three-day sleep patterns in *Drosophila*. (A) Sleep profiles of female and male flies under LD and DD condition for three days (day-after-eclosion, DAE, 5 to 7). Before recording, virgin male or female flies were sorted by gender separately since DAE 1, and raised in SA medium from DAE 2 to 4 at LD condition. Left for female and right for male. (B) Comparison of total sleep for three consecutive days under LD and DD condition. N numbers: females in LD, females in DD, males in LD, and males in DD: 16, 11, 14, and 11. Statistical methods are the same as in Fig 1.
(TIF)

S2 Fig Portfolios of drug effects on *Drosophila* sleep from ZT 12–24. The 12-hour results in prior to Figs 4 and 5 were summarized. Changes of individual parameters were normalized against the vehicle group. (A) Total sleep, (B) Mean sleep, (C) Maximum sleep, (D) Sleep frequency, and (E) Activity index. For N numbers: vehicle, N = 93; melatonin, N = 15; pentobarbital, N = 16; phenobarbital, N = 12; *F*. *multiflora*, N = 26; *G*. *uralensis*, N = 14; *M*. *officinalis*, N = 13; *P*. *ginseng*, N = 14; *P*. *notoginseng*, N = 28; *P*. *vulgaris*, N = 15; *W*. *somnifera*, N = 13. Statistical methods are the same as in Fig 4.
(TIF)

S3 Fig Sleep profiles for AD and control male flies. (A) Typical sleep profiles for male AD flies under LD or DD condition. (B) Summary of total sleep time at specific time periods. N numbers for control and AD were 102 and 56, respectively. Statistical methods are the same as in Fig 1.
(TIF)

S4 Fig Raw data for sleep parameters in female AD flies. Figs A-F illustrated the original data in female AD and control flies before normalization, as shown in Fig 6. Statistical methods are the same as in Fig 1.
(TIF)
