# Supplementary figures and images for: Screening of sleep assisting drug candidates with a Drosophila model
Source: PLoS One. 2020 Jul 29;15(7):e0236318. doi: 10.1371/journal.pone.0236318 (PMC7390450; doi:10.1371/journal.pone.0236318)

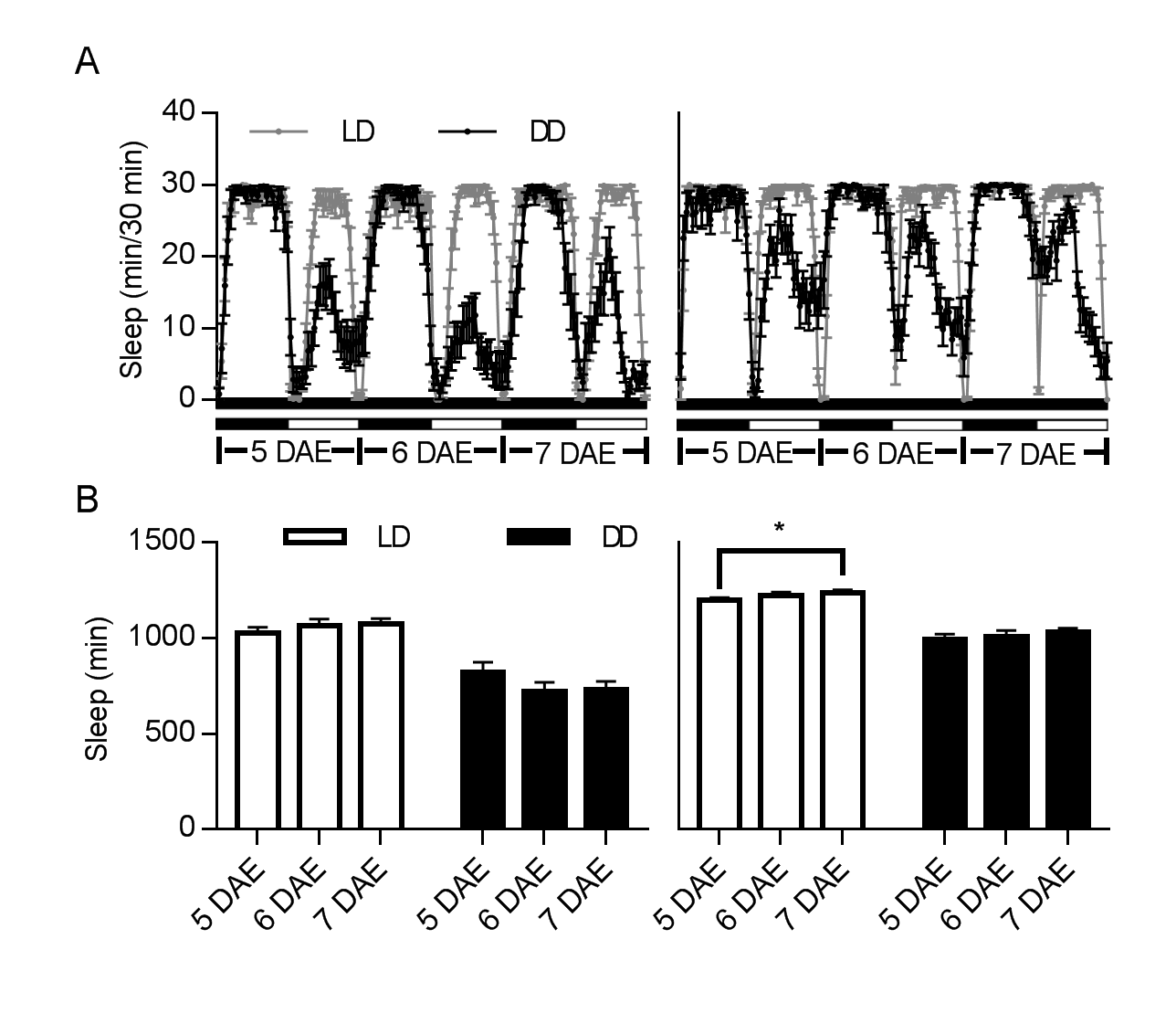

Supplement: S1 Fig — (A) Sleep profiles of female and male flies under LD and DD condition for three days (day-after-eclosion, DAE, 5 to 7). Before recording, virgin male or female flies were sorted by gender separately since DAE 1, and raised in SA medium from DAE 2 to 4 at LD condition. Left for female and right for male. (B) Comparison of total sleep for three consecutive days under LD and DD condition. N numbers: females in LD, females in DD, males in LD, and males in DD: 16, 11, 14, and 11. Statistical methods are the same as in Fig 1. (TIF) [file pone.0236318.s001.tif]

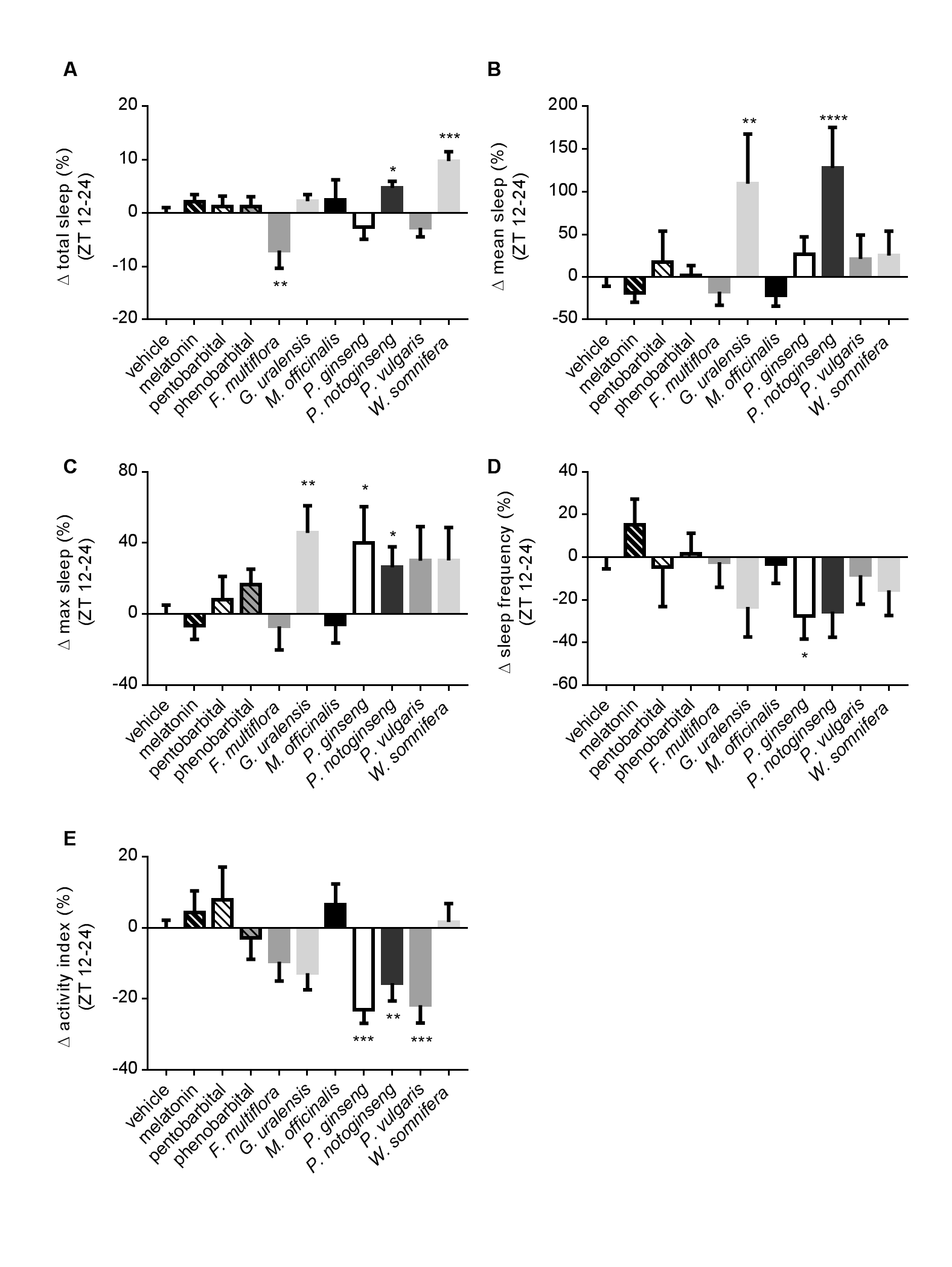

Supplement: S2 Fig — The 12-hour results in prior to Figs 4 and 5 were summarized. Changes of individual parameters were normalized against the vehicle group. (A) Total sleep, (B) Mean sleep, (C) Maximum sleep, (D) Sleep frequency, and (E) Activity index. For N numbers: vehicle, N = 93; melatonin, N = 15; pentobarbital, N = 16; phenobarbital, N = 12; F. multiflora, N = 26; G. uralensis, N = 14; M. officinalis, N = 13; P. ginseng, N = 14; P. notoginseng, N = 28; P. vulgaris, N = 15; W. somnifera, N = 13. Statistical methods are the same as in Fig 4. (TIF) [file pone.0236318.s002.tif]

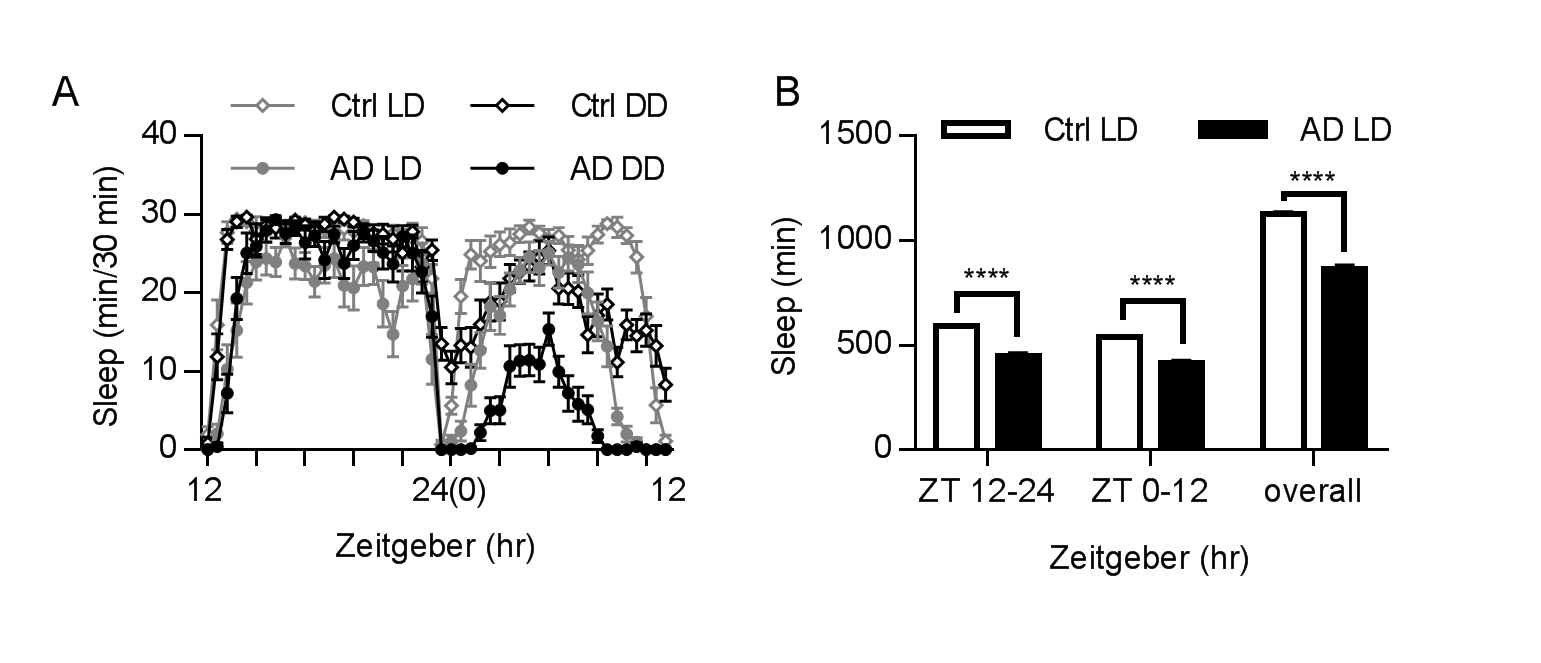

Supplement: S3 Fig — (A) Typical sleep profiles for male AD flies under LD or DD condition. (B) Summary of total sleep time at specific time periods. N numbers for control and AD were 102 and 56, respectively. Statistical methods are the same as in Fig 1. (TIF) [file pone.0236318.s003.tif]

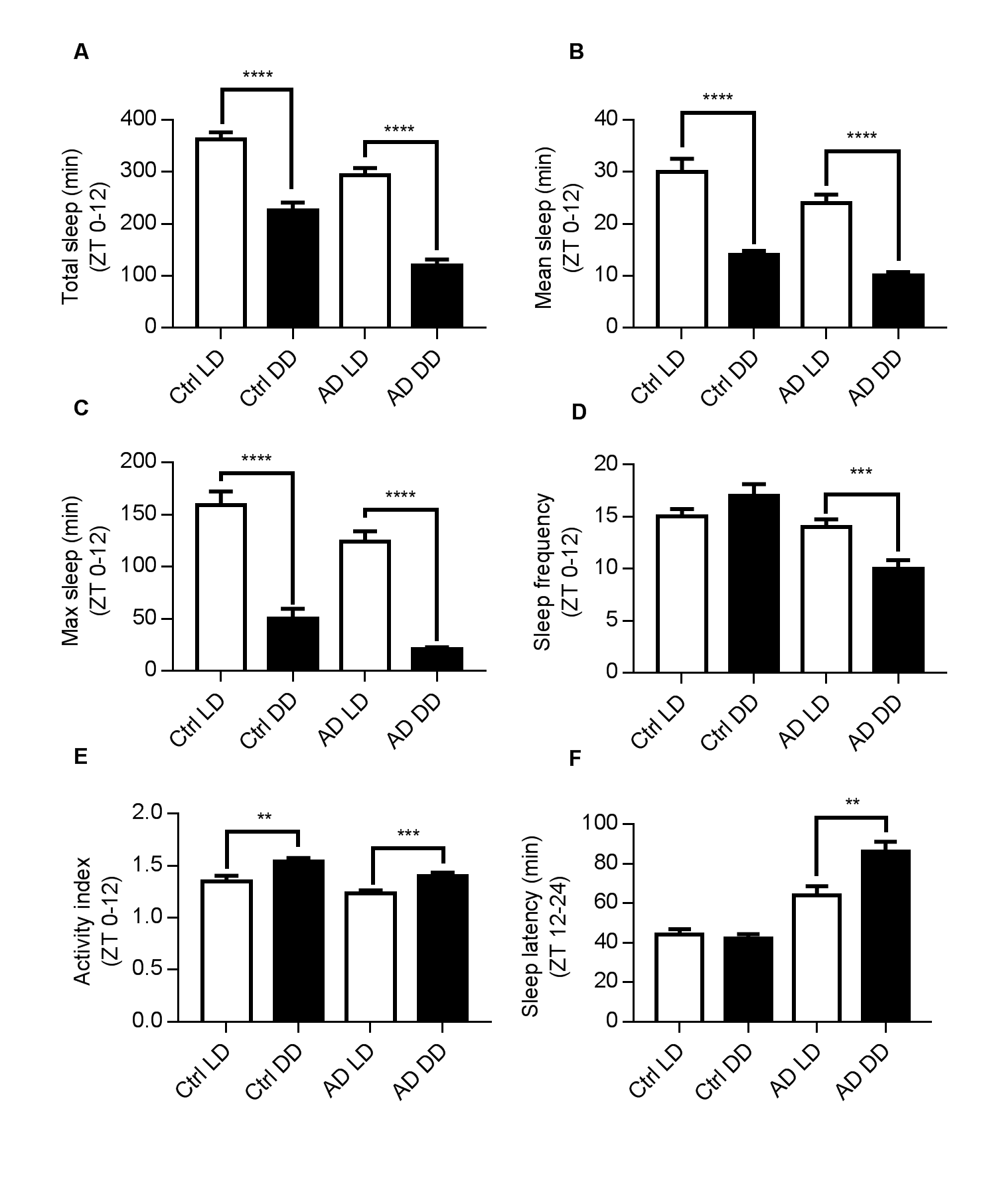

Supplement: S4 Fig — Figs A-F illustrated the original data in female AD and control flies before normalization, as shown in Fig 6. Statistical methods are the same as in Fig 1. (TIF) [file pone.0236318.s004.tif]
